# Curcumin slows osteoarthritis progression and relieves osteoarthritis-associated pain symptoms in a post-traumatic osteoarthritis mouse model

**DOI:** 10.1186/s13075-016-1025-y

**Published:** 2016-06-03

**Authors:** Zhuo Zhang, Daniel J. Leong, Lin Xu, Zhiyong He, Angela Wang, Mahantesh Navati, Sun J. Kim, David M. Hirsh, John A. Hardin, Neil J. Cobelli, Joel M. Friedman, Hui B. Sun

**Affiliations:** Department of Orthopaedic Surgery, Albert Einstein College of Medicine, Bronx, NY USA; Department of Radiation Oncology, Albert Einstein College of Medicine, Bronx, NY USA; China-Japan Union Hospital of Jilin University, Bronx, NY USA; Department of Physiology & Biophysics, Albert Einstein College of Medicine, Bronx, NY USA; Department of Medicine, Albert Einstein College of Medicine, Bronx, NY USA

**Keywords:** Post-traumatic osteoarthritis, Curcumin, Nutraceuticals, Chondroprotection, MMPs, ADAMTS5

## Abstract

**Background:**

Curcumin has been shown to have chondroprotective potential in vitro. However, its effect on disease and symptom modification in osteoarthritis (OA) is largely unknown. This study aimed to determine whether curcumin could slow progression of OA and relieve OA-related pain in a mouse model of destabilization of the medial meniscus (DMM).

**Methods:**

Expression of selected cartilage degradative-associated genes was evaluated in human primary chondrocytes treated with curcumin and curcumin nanoparticles and assayed by real-time PCR. The mice subjected to DMM surgery were orally administered curcumin or topically administered curcumin nanoparticles for 8 weeks. Cartilage integrity was evaluated by Safranin O staining and Osteoarthritis Research Society International (OARSI) score, and by immunohistochemical staining of cleaved aggrecan and type II collagen, and levels of matrix metalloproteinase (MMP)-13 and ADAMTS5. Synovitis and subchondral bone thickness were scored based on histologic images. OA-associated pain and symptoms were evaluated by von Frey assay, and locomotor behavior including distance traveled and rearing.

**Results:**

Both curcumin and nanoparticles encapsulating curcumin suppressed mRNA expression of pro-inflammatory mediators IL-1β and TNF-α, MMPs 1, 3, and 13, and aggrecanase ADAMTS5, and upregulated the chondroprotective transcriptional regulator CITED2, in primary cultured chondrocytes in the absence or presence of IL-1β. Oral administration of curcumin significantly reduced OA disease progression, but showed no significant effect on OA pain relief. Curcumin was detected in the infrapatellar fat pad (IPFP) following topical administration of curcumin nanoparticles on the skin of the injured mouse knee. Compared to vehicle-treated controls, topical treatment led to: (1) reduced proteoglycan loss and cartilage erosion and lower OARSI scores, (2) reduced synovitis and subchondral plate thickness, (3) reduced immunochemical staining of type II collagen and aggrecan cleavage epitopes and numbers of chondrocytes positive for MMP-13 and ADAMTS5 in the articular cartilage, and (4) reduced expression of adipokines and pro-inflammatory mediators in the IPFP. In contrast to oral curcumin, topical application of curcumin nanoparticles relieved OA-related pain as indicated by reduced tactile hypersensitivity and improved locomotor behavior.

**Conclusion:**

This study provides the first evidence that curcumin significantly slows OA disease progression and exerts a palliative effect in an OA mouse model.

**Electronic supplementary material:**

The online version of this article (doi:10.1186/s13075-016-1025-y) contains supplementary material, which is available to authorized users.

## Background

Osteoarthritis (OA) is a progressive and degenerative disease of the articular joints involving the articular cartilage, synovium, and subchondral bone, and is a leading cause of pain and disability in the adult population [1]. Despite the high prevalence of OA, there is currently no cure or effective treatment that halts or reverses disease progression [2]. While current pharmacologic treatments such as analgesics and nonsteroidal anti-inflammatory drugs (NSAIDs) provide symptomatic relief, such as relieving pain, they do not exert a clear clinical effect on OA disease prevention or modification [3]. In most cases, long-term use of these treatments has been associated with substantial gastrointestinal, renal, and cardiovascular side effects [3]. There is a clear and urgent need for new therapeutic strategies that are effective and safe for OA treatment.

Curcumin, the principal curcuminoid and the most active component in turmeric, is a biologically active phytochemical [4, 5]. Evidence from several recent in vitro studies suggests that curcumin may exert a chondroprotective effect through actions such as anti-inflammatory, anti-oxidative stress, and anti-catabolic activity that are critical for mitigating OA disease pathogenesis and symptoms. For example, curcumin has been shown to mitigate the inflammatory process by decreasing synthesis of inflammatory mediators such as interleukin (IL)-1β, tumor necrosis factor (TNF)-α, IL-6, IL-8, prostaglandin E2 (PGE_2_)_,_ and cyclooxygenase-2 (COX-2) [6–8], inhibit IL-1β-induced extracellular matrix degradation [9] and chondrocyte apoptosis [10, 11], and mitigate the over-production of reactive oxygen and nitrogen species [12, 13]. Moreover, curcumin, by inhibiting the activator protein 1 (AP-1) pathway [14] and nuclear factor kappa B (NF-kB) activation [14–16], suppresses the gene expression of a number of matrix metalloproteinases (MMPs), which play critical roles in the breakdown of the cartilage extracellular matrix [7, 14–17].

Despite the recent progress, the effect of curcumin on OA disease progression and pain relief is largely unknown. Moon et al. showed that following intraperitoneal injection of curcumin every other day for 2 weeks, expression of TNF-α and IL-1β in the ankle joint, and serum immunoglobulin concentrations in mice with collagen-induced arthritis were downregulated compared with non-curcumin-treated mice [18], suggesting curcumin may be beneficial in rheumatoid arthritis. Furthermore, Colitti et al. found that oral delivery of curcumin in canines with spontaneous OA leads to decreased IL-18 and TNF-α production, and inhibition of the inflammatory transcription factor NF-kB in white blood cells [19]. The study suggests a potential anti-inflammatory effect of curcumin on the joints in OA.

While several studies suggest oral administration of curcumin may exert an effect in relieving OA-related pain [20–24], topical application may provide another patient-friendly method of treatment. Importantly, it may increase the bioavailability of curcumin at the disease site for OA treatment. In this study, we aim to determine the efficacies of curcumin through oral delivery and custom-made nanoparticles through topical administration in OA disease and symptom modification using a mouse model of post-traumatic OA.

## Methods

### Cell culture and curcumin treatment in vitro

All human studies were approved by the Albert Einstein College of Medicine Institutional Review Board. Human primary chondrocytes derived from patients undergoing joint replacement surgery (women aged 58–69 years, n = 3) were cultured in DMEM/F12 with 10 % fetal bovine serum [25]. Prior to curcumin treatment, cells were cultured in the DMEM/F12 with 1 % fetal bovine serum overnight. In some experiments, chondrocytes were incubated with IL-1β (10 ng/ml, Sigma) 30 minutes prior to incubation with curcumin (100 μM, Sigma) or curcumin (100 μM) encapsulated within nanoparticles for 6 hours. Cells were then lysed and RNA isolated for reverse transcription-quantitative polymerase chain reaction (real-time-PCR) [26]. The dose (100 μM) and treatment duration (6 hours) were chosen based on assays for dose-response (0–200 μM) and time course (0–48 hours) of human primary chondrocytes treated with non-encapsulated curcumin (Additional file 1: Figure S1).

### Preparation of curcumin nanoparticles

Curcumin nanoparticles were prepared using a variation of a nanoparticle platform that was developed for topical and systemic delivery of nitric oxide [27–29] in three steps as follows [30]: (1) hydrolysis of tetra-methyl-orthosilicate (TMOS). Hydrolyzed TMOS is prepared by sonicating at ice temperature, a mixture of 3 ml TMOS and 600 μl 1 mM HCl in a small glass bottle with rubber stopper. Upon sonication the initial biphasic solution turns into a monophasic solution. The monophasic solution is stored at 4 °C for an hour to help eliminate methanol, a byproduct of TMOS hydrolysis (residual methanol is further eliminated during the lyophilization process); (2) polymerization. The following ingredients are added sequentially to a 50-ml conical tube, which is inverted (to facilitate mixing) after each addition of an ingredient: 24 ml of PBS 50 mM pH 7.5, 1.5 ml PEG 400, 1.5 ml chitosan (5 mg/ml) at pH 6 in acetic acid, 4 ml of 5 mg/ml curcumin (Sigma) dissolved in dimethyl sulfoxide (DMSO) and finally 3 ml hydrolyzed TMOS. After all the ingredients are mixed a homogeneous gel is formed in approximately 30 minutes; (3) lyophilization and ball-milling. The wet sol-gel containing the curcumin is freeze-dried overnight. The resulting dry course powder is then ball-milled and stored in a sealed vial for subsequent use for the experiments. A very similar version of this platform has been used to treat topical infections and accelerate wound healing [31].

### Induction of osteoarthritis in mice and curcumin treatment

All animal studies were approved by the Albert Einstein College of Medicine Institutional Animal Care and Use Committee. Destabilization of the medial meniscus (DMM) was established in adult C57BL/6 male mice (male, 5–6 months of age) by surgically transecting the medial meniscotibial ligament (MMTL) in the right hind limb [32]. Briefly, the joint capsule immediately medial to the patellar tendon was incised, followed by blunt dissection of the infrapatellar fat pad, to provide visualization of the MMTL of the medial meniscus. The MMTL was transected, leading to destabilization of the medial meniscus (DMM). In the sham surgery, the MMTL was visualized but not transected. The joint capsule and skin were closed by suture. Immediately after the DMM surgery, mice were subjected to (1) oral administration of 50 mg/kg curcumin (Sigma) dissolved in corn oil or vehicle (corn oil only) administered via oral gavage (n = 8/group), or (2) topical application of curcumin nanoparticles (0.07 mg of 10 μg curcumin/1 mg nanoparticles) or vehicle control (coconut oil) on the skin, within a 5-mm^2^ area directly above the DMM-operated knee (n = 5/group), once daily for 8 weeks.

### Safranin O staining, OARSI score, and histologic evaluation of synovium and subchondral bone

Animals were sacrificed at 8 weeks following curcumin treatment. The hind limbs were fixed in formalin, decalcified in formic acid, embedded in paraffin, and sectioned for histological and immunohistochemical analysis. afranin O-fast green staining was used to visualize proteoglycans in the articular cartilage. The severity of OA was evaluated in the medial compartment of the knee with at least five sections for each mouse using the Osteoarthritis Research Society International (OARSI) scoring system [33]. The synovial pathology (i.e., synovitis) was analyzed on Safranin O stained sections from which the OARSI scores were obtained. The degree of synovitis was scored using a scoring system that measured the thickness of the synovial lining cell layer on a scale of 0–3 (0 = 1–2 cells, 1 = 2–4 cells, 2 = 4–9 cells and 3 = 10 or more cells) and cellular density in the synovial stroma on a scale of 0–3 (0 = normal cellularity, 1 = slightly increased cellularity, 2 = moderately increased cellularity and 3 = greatly increased cellularity). Synovitis scores obtained from all four quadrants (medial tibia, medial femur, lateral tibia, and lateral femur) for both of the above parameters were averaged separately and then the sum of averages from both parameters was used for analysis (on a scale of 0–6) [34]. The thickness of the medial subchondral bone plate (region between the osteochondral junction and marrow space on the medial side of the tibial plateau, in μm) was measured using AxioVision software using Safranin O stained sections from which the OARSI and synovitis scores were obtained [35].

### Immunohistochemical analysis

Sections were incubated overnight at 4 °C with antibodies against cleaved aggrecan (NITEGE, Ibex) and cleaved type II collagen (Col2-3/4 M, Ibex), matrix metalloproteinase (MMP)-13 (Abcam), and a disintegrin and metalloproteinase with thrombospondin motifs (ADAMTS)5 (Abcam) followed by incubation with anti-mouse or anti-rabbit secondary antibody (Biocare Medical) and visualization with 3,3-diaminobenzidine (DAB) chromagen (Vector Laboratories). Negative controls were stained with irrelevant isotype-matched antibodies (Biocare Medical). Immunostaining intensity for type II collagen or aggrecan cleavage epitopes was quantified by determining the “reciprocal intensity” of the stained articular cartilage matrix; briefly, the light intensity value of six random locations within all three zones from the posterior to anterior direction of the femoral and tibial condyles of three sections per mouse was measured using the color picker in Adobe Photoshop [36, 37]. Percentages of positive MMP-13 and ADAMTS5 chondrocytes were determined by counting the number of immunostained cells and dividing by the total number of chondrocytes visualized by a hematoxylin counterstain (Vector Laboratories).

### In vivo localization of topically applied curcumin

Curcumin nanoparticles (0.07 mg of 10 μg curcumin/1 mg nanoparticles dissolved in coconut oil) or vehicle control (coconut oil) were topically applied on the right knee of adult C57BL/6 mice (male, 5–6 months). At 3, 6, and 24 hours after treatment (n = 3/group), the animals were sacrificed and the hind limbs were fixed in formalin, decalcified in formic acid, embedded in paraffin and sectioned for histological analysis. Sections (5-μm) were stained with hematoxylin and eosin (H&E), and imaged with confocal microscopy to localize the curcumin particles within the articular joint. In a separate group of animals, mice were sacrificed 3 hours after topical application of curcumin nanoparticles or vehicle control (n = 3/group). The IPFP from the right knee was dissected and flash frozen. RNA was isolated for real-time PCR.

### von Frey testing

Mice were acclimated for 30 minutes in individual chambers on top of a wire grid platform prior to von Frey testing. The plantar surface of the hind paw was stimulated with ascending force intensities of von Frey filaments (Stoelting) to determine tactile sensitivity. A positive response was defined as a rapid withdrawal of the hind paw when the stimulus was applied, and the number of positive responses for each stimulus was recorded. Tactile threshold was defined as a withdrawal response in 5 out of 10 trials to a given stimulus intensity [37]. This threshold was calculated once per animal.

### Pain and OA-related behavioral tests

As we and others previously described, mice were acclimated to the test room for 30 minutes before open field testing [37, 38]. Mice were placed in the center of individual plexiglass square chambers (45 cm × 45 cm) and allowed to freely explore the chamber for the duration of the 6-minute test session. The movements of the mice were recorded with a video camera. Upon completion of the test, which was performed once per animal, each mouse was returned to its home cage. Two observers blinded to treatment group assignments manually traced mouse movements to calculate the distance (in cm) that the mouse traveled within the cage in 6 minutes (distance traveled), and recorded the number of times each mouse reared (standing on its hind limbs) within 6 minutes (rearing) [38].

### Real-time PCR

Total RNA was isolated with an RNeasy kit (Qiagen) and cDNA was synthesized using the iScript Reverse Transcriptase kit (Bio-Rad). SYBR Green real-time PCR (Bio-Rad) was performed in duplicate for each sample to determine relative gene expression using *Glyceraldehyde-3-phosphate dehydrogenase* (*GAPDH*) as a housekeeping control with the 2^-ΔΔCt^ method [26, 37].

### Statistical analysis

Results are expressed as mean ± SD. Significance was determined using Student’s *t* test or one-way analysis of variance (ANOVA) and Tukey’s multiple comparison test with a significance level of *p* < 0.05 (GraphPad).

## Results

### Gene expression profile change favors chondroprotection in curcumin-treated human chondrocytes in vitro

**Fig. 1:**
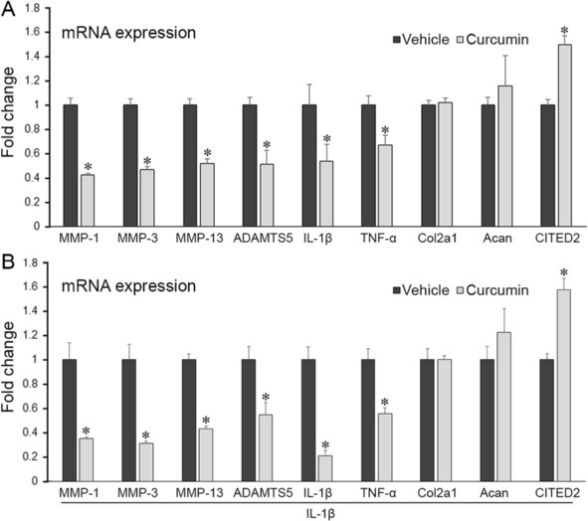
Chondrocytes treated with curcumin exhibits a gene expression profile that is favorable for chondroprotection. Human primary chondrocytes treated with curcumin for 6 hours exhibited reduced mRNA levels of matrix metalloproteinase (*MMP*)-1, MMP-3, MMP-13, a disintegrin and metalloproteinase with thrombospondin motifs (*ADAMTS*)5, IL-1β, TNF-α, and increased CITED2 compared to vehicle-treated cells, in the absence (**a**) and presence (**b**) of IL-1β, while expression of collagen 2a1 (*Col2a1*) and aggrecan (*Acan*) remained unchanged. *P < 0.05, *t* test, n = 3/group

We first validated the chondroprotective potential of curcumin by gene expression profile analysis in chondrocytes in vitro. Consistent with previous studies [7, 14–17], human primary chondrocytes, in the absence and presence of IL-1β, and treated with curcumin, exhibited significantly reduced mRNA levels of proteolytic enzymes MMP-1, MMP-3, and MMP-13, and pro-inflammatory cytokines IL-1β and TNF-α (*p* < 0.05) (Fig. 1). Interestingly, as shown for the first time, curcumin significantly reduced expression of aggrecanase ADAMTS5 and increased expression of CITED2 (Cbp/p300 Interacting Transactivator with ED-rich tail 2), MMP-repressing transcriptional regulator (*p* < 0.05) (Fig. 1). No effects of curcumin on expression of anabolic genes collagen 2a1 and aggrecan were observed (*p* > 0.05) (Fig. 1).

### Oral delivery of curcumin slows disease progression but does not significantly affect OA-related symptoms in mice with DMM

**Fig. 2:**
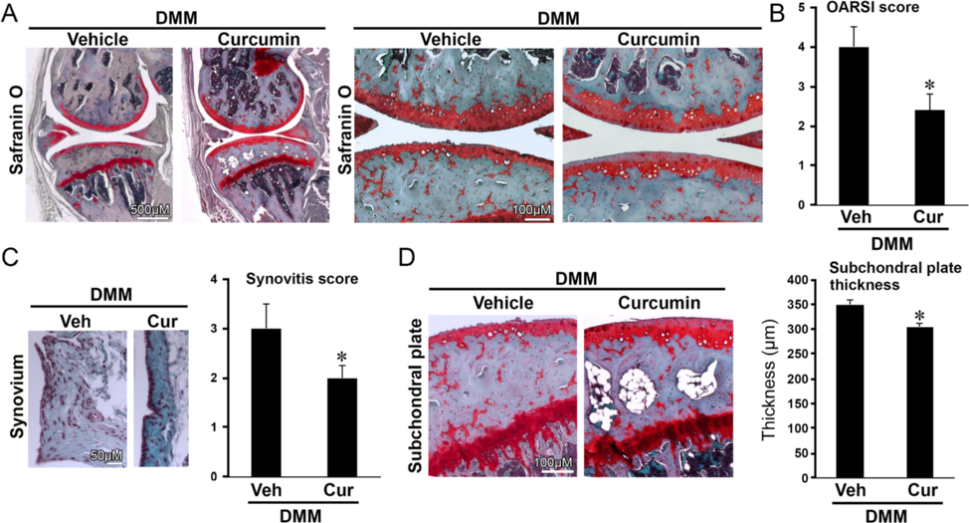
Oral administration of curcumin slowed progression of post-traumatic osteoarthritis in mice. Mice with destabilization of the medial meniscus (*DMM*) were treated daily with curcumin (*Cur*) or vehicle via oral gavage. Mice with DMM treated with curcumin exhibited improved Safranin O staining (**a**), lower Osteoarthritis Research Society International (*OARSI*) scores (**b**), and reduced synovitis (**c**) and subchondral plate thickness (**d**) at 8 weeks following surgery, compared to mice with DMM that were treated with vehicle (*Veh*). *P < 0.05, *t* test, n = 8/group. Representative histologic images are shown

We next determined the efficacy of curcumin on DMM-induced OA through oral administration by evaluating the structural integrity of the articular cartilage using microscopy following Safranin O staining and OARSI evaluation. Eight weeks after DMM, the articular cartilage in the limb with DMM in the vehicle-treated mice exhibited moderate pathological osteoarthritic changes characterized by Safranin O loss, cartilage fibrillation, and cartilage erosion (Fig. 2a), with an OARSI score of 4.0 ± 0.5. (Fig. 2b). In contrast, the cartilage in the limb with DMM in curcumin-treated mice exhibited less Safranin O loss and cartilage fibrillation (Fig. 2a) with a significantly lower OARSI score (2.4 ± 0.42) compared to that in vehicle-treated controls (*p* < 0.05, Fig. 2b). Curcumin treatment also significantly reduced synovitis (Fig. 2c) and subchondral plate thickness (Fig. 2d) compared to vehicle controls (*p* < 0.05 for both). However, oral administration of curcumin had no significant effect on mitigating OA-related pain, as evaluated by von Frey testing, distance traveled and hind limb rearing (not shown).

### Curcumin nanoparticles exert an anti-catabolic and anti-inflammatory effect in human chondrocytes in vitro

**Fig. 3:**
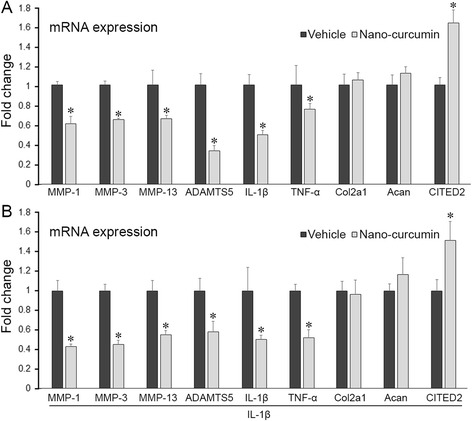
Curcumin nanoparticles exert anti-catabolic and anti-inflammatory effect on gene expression of human primary chondrocytes in the absence of IL-1β (**a**) and presence of IL-1β (**b**). Human primary chondrocytes treated with nano-encapsulated curcumin (nano-curcumin) for 6 hours exhibited reduced mRNA levels of matrix metalloproteinase (*MMP*)-1, MMP-3, MMP-13, a disintegrin and metalloproteinase with thrombospondin motifs (*ADAMTS5*), IL-1β, TNF-α, and increased levels of CITED2 compared to that in vehicle-treated cells, while expression of collagen 2a1 (*Col2a1*) and aggrecan (*Acan*) remained unchanged. *P < 0.05, *t* test, n = 5/group

While oral administration of non-encapsulated curcumin exhibited significant efficacy in slowing the progression of OA, its therapeutic efficacy may be restricted by its relatively poor oral bioavailability [39]. We therefore developed curcumin nanoparticles using a novel polymeric nanoparticle carrier [30]. To test whether nanoparticles encapsulating curcumin affect the chondroprotective potential of curcumin, we compared the gene expression profile in primary cultured human chondrocytes in the absence or presence of IL-1β and treated with curcumin nanoparticles or vehicle control. Curcumin nanoparticles significantly reduced mRNA levels of MMP-1, MMP-3, MMP-13, ADAMTS5, IL-1β and TNF-α, and increased levels of CITED2 chondrocytes compared to the vehicle control (*p* < 0.05 for all), at a comparable level to that of non-encapsulated curcumin-treated chondrocytes, based on the equivalent concentration of curcumin, in the absence or presence of IL-1β (*p* > 0.05 for all, Fig. 3). No significant effects of curcumin nanoparticles on expression of collagen 2a1 and aggrecan were observed (*p* > 0.05) (Fig. 3).

### Topical curcumin nanoparticles localize and are effective in the infrapatellar fat pad (IPFP)

**Fig. 4:**
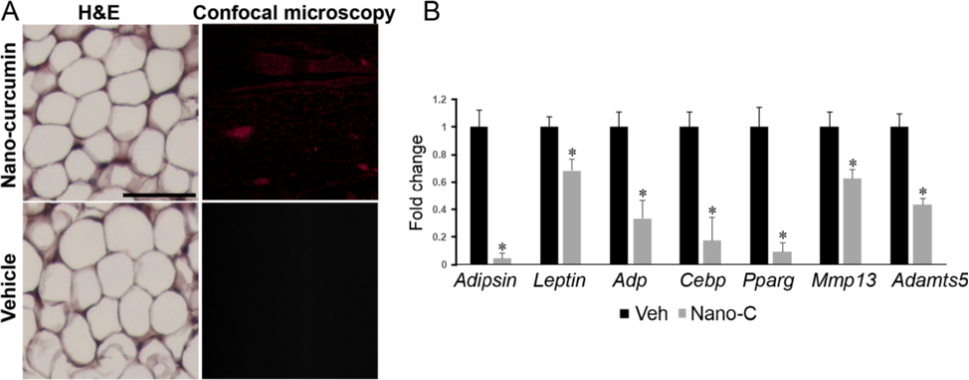
Topical application of curcumin nanoparticles on the mouse knee is localized in the infrapatellar fat pad and suppresses adipokine and adipogenesis-related gene expression. H&E staining and confocal microscopy of the infrapatellar fat pad of mouse knee joints treated with topical nano-encapsulated curcumin (*Nano-C*). Representative histologic images are shown (**a**). Relative mRNA expression of adipokines, adipogenesis-related transcription regulators, matrix metalloproteinase (*MMP*-*13*), and a disintegrin and metalloproteinase with thrombospondin motifs (*ADAMTS5*) in the infrapatellar fat pad of curcumin nanoparticles-treated mice vs vehicle controls (**b**). *Adp* Adiponectin, *Pparg* peroxisome proliferator-activated receptor gamma, *Cebpa* CCAAT/enhancer binding protein alpha, *Veh* vehicle. *P < 0.05, *t* test, n *=* 3/group*. Scale bar* = 50 μm

To test whether local, topical application of curcumin nanoparticles would exert increased efficacy in treating OA, we first determined whether curcumin nanoparticles could penetrate into the joint tissues following topical application to the mouse knee. Curcumin was detected in the IPFP at 3 hours following topical application as shown in Fig. 4a, but was not detected in the articular cartilage or other joint tissues, or at 6 or 24 hours following topical application (not shown), using confocal microscopy based on the auto-fluorescence of curcumin [40]. As curcumin was localized within the IPFP, we next examined the effect of curcumin nanoparticle topical treatment on the gene expression profile of pro-inflammatory mediators in the IPFP, which have been shown to have a significant impact on cartilage homeostasis and OA [41–43]. As revealed by real-time PCR, the treatment suppressed mRNA expression of adipokines *adipsin*, *leptin*, *adiponectin*, adipo-regulatory transcription factors CCAAT/enhancer binding protein alpha (*Cebpa*) and peroxisome proliferator-activated receptor gamma (*Pparg*), and *Mmp13* and *Adamts5* (*p* < 0.05 for all, Fig. 4b).

### Topical application of curcumin nanoparticles slows progression of OA in mice with DMM

**Fig. 5:**
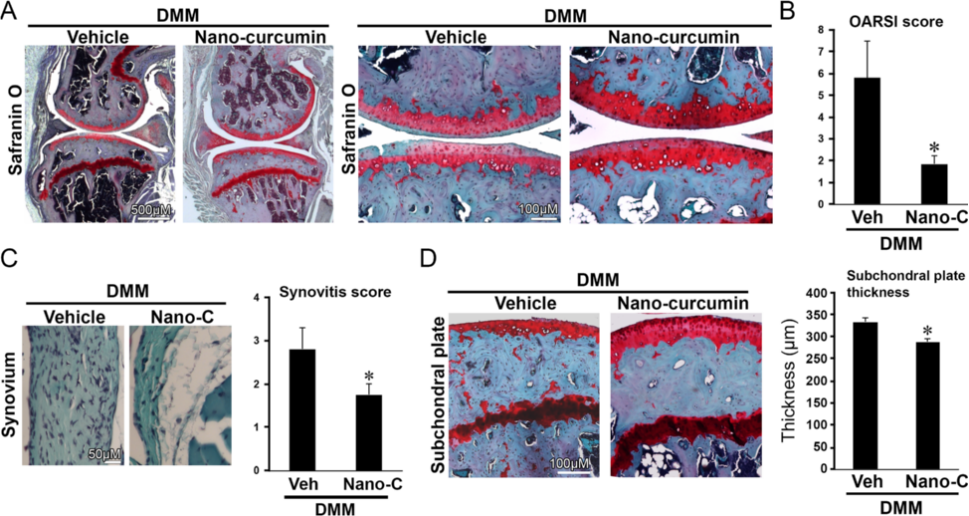
Topical application of nano-encapsulated curcumin slowed the progression of OA induced by destabilization of the medial meniscus (*DMM*) in mice. Mice with DMM were treated daily with topical application of curcumin nanoparticles or vehicle. Mice treated topically with curcumin nanoparticles (*Nano-C*) exhibited improved Safranin O staining (**a**), lower Osteoarthritis Research Society International (*OARSI*) scores (**b**), and reduced synovitis (**c**), and subchondral bone plate thickness (**d**) at 8 weeks after surgery compared to that in vehicle control (*Veh*) (*p < 0.05, *t* test, n = 5/group). Representative histologic images are shown

To determine efficacy of topical application of curcumin nanoparticles on OA disease progression, we evaluated structural integrity of the articular cartilage after eight weeks of daily topical curcumin treatment beginning immediately following DMM in mice. Eight weeks after DMM, the articular cartilage in the limb with DMM in the vehicle-treated mice exhibited moderate pathological osteoarthritic change characterized by Safranin O loss and cartilage fibrillation (Fig. 5a), and an average OARSI score of 5.8 ± 2.1 (Fig. 5b). In contrast, the cartilage in the limb with DMM in mice treated with curcumin nanoparticles exhibited less Safranin O loss and cartilage fibrillation (Fig. 5a), and the mean OARSI score (1.8 ± 0.35) was significantly lower compared to vehicle-treated controls (*p* < 0.05, Fig. 5b). In addition, curcumin nanoparticles significantly reduced synovitis (Fig. 5c) and subchondral plate thickness (Fig. 5d) compared to vehicle-treated controls (*p* < 0.05 for both).

### Topical application of curcumin nanoparticles reduced matrix degradation markers and levels of MMP-13 and ADAMTS5 in cartilage from mice with DMM

**Fig. 6:**
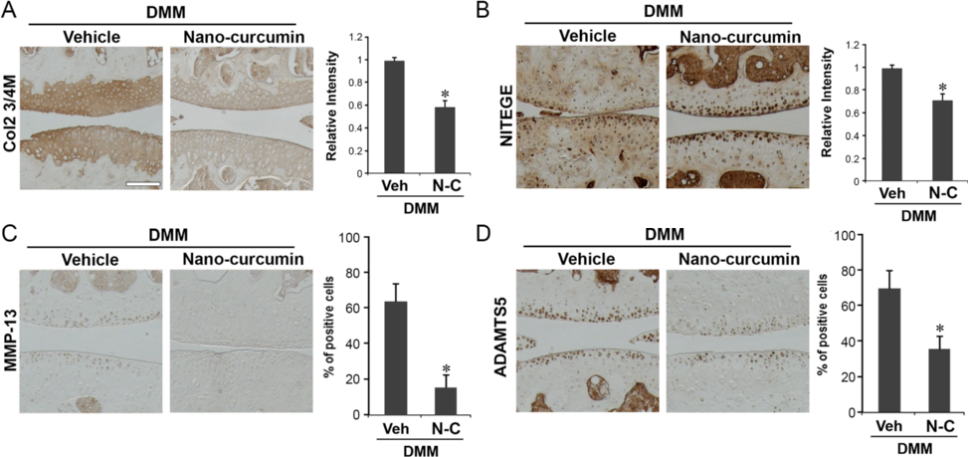
Topical application of curcumin nanoparticles reduced the degradation of articular cartilage matrix and reduced the expression of matrix metalloproteinase-13 (*MMP-13*) and a disintegrin and metalloproteinase with thrombospondin motifs-5 (*ADAMTS5*). Intensity of immunohistochemical staining of type II collagen cleavage epitope (*Col2-3/4 M*) (**a**) and cleaved aggrecan (*NITEGE*) (**b**), and percentage of positive cells of immunohistochemical staining of MMP-13 (**c**), and of ADAMTS5 (**d**) in the articular cartilage of mice with destabilization of the medial meniscus (*DMM*) that were treated with curcumin nanoparticles (*N-C*) for 8 weeks following surgery were significantly reduced compared to mice with DMM treated with vehicle (*Veh*) (*p < 0.05, *t* test, n = 5/group). *Scale bar* = 100 μM. Representative immunohistochemical images are shown

Immunohistochemical staining showed that topical curcumin treatment strongly reduced the levels of the type II collagen cleavage epitope (Col2 3/4 M) in mice with DMM compared to vehicle-treated mice with DMM (Fig. 6a). Based on the immunostaining intensities of six randomly selected areas of the articular cartilage at 8 weeks following DMM, type II collagen cleavage was reduced to 0.58-fold in curcumin-treated animals compared to vehicle-treated controls (*p* < 0.05, Fig. 6a). Immunohistochemical staining similarly showed that curcumin nanoparticle treatment reduced the levels of cleaved aggrecan (NITEGE) in mice with DMM compared to vehicle-treated mice with DMM at 8 weeks (Fig. 6b). At 8 weeks after DMM, the immunostaining intensity of cleaved aggrecan in curcumin nanoparticle-treated mice with DMM was reduced to 0.68-fold compared to vehicle-treated mice (*p* < 0.05, Fig. 6b).

Cartilage matrix degradation is mainly mediated by two major families of proteolytic enzymes, namely MMPs and ADAMTS [44]. In particular, MMP-13 is the most potent enzyme in cleaving type II collagen, the principal form in articular cartilage, while ADAMTS5 has been shown in mice to cleave aggrecan, the major cartilage proteoglycan [2]. We therefore examined whether reduction of MMP-13 and ADAMTS5 could underlie the chondroprotective effect of curcumin using immunohistochemical analysis.

At 8 weeks following DMM, the percentage of MMP-13-positive cells in the articular cartilage was reduced from 63 % in vehicle-treated mice to 16 % in curcumin-treated mice (*p* < 0.05, Fig. 6c). Similarly, curcumin reduced the percentage of ADAMTS5-positive cells from 68 % in vehicle-treated mice to 37 % (*p* < 0.05, Fig. 6d). These data suggest that curcumin treatment improves the integrity of the articular cartilage by preserving both collagen and aggrecan components in mice with post-traumatic OA, and that the chondroprotective effects exerted by curcumin are mediated, at least in part, by suppressing the predominant collagenase MMP-13 and predominant aggrecanase ADAMTS5.

### Topical curcumin nanoparticles reduce OA-related pain

**Fig. 7:**
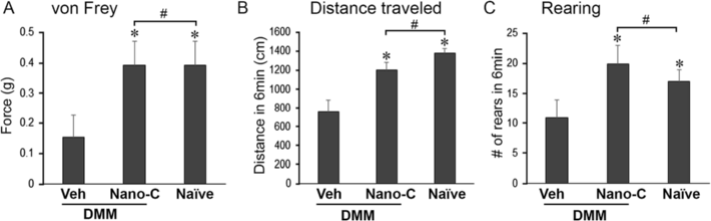
Topical application of curcumin nanoparticles reduces osteoarthritis-related pain symptoms. Tactile sensitivity (von Frey testing) (**a**), and distance traveled (**b**) and number of times reared (**c**) per 6 minutes in an open field, in mice with destabilization of the medial meniscus (*DMM*) treated with curcumin nanoparticles (*Nano-C*) at 8 weeks after DMM surgery, did not differ from naïve controls (#*p* > 0.05, one-way analysis of variance (ANOVA) with Tukey post-hoc test, n = 5/group), but had significant improvement compared to mice with DMM treated with vehicle (*Veh*) (*p < 0.05, one-way ANOVA with Tukey post-hoc test, n = 5/group)

The progression of OA is accompanied by secondary clinical symptoms, most prominently pain [45, 46]. At 8 weeks following DMM, vehicle-treated mice exhibited reductions in the threshold of response to mechanical stimuli (*p* < 0.05, von Frey assay, Fig. 7a), distance traveled (Fig. 7b), and rearing (standing on hind limbs, Fig. 7c), compared to naïve controls (*p* < 0.05). Animals topically treated with curcumin nanoparticles exhibited reduced tactile hypersensitivity (*p* > 0.05, Fig. 7a), and increased distance traveled (*p* > 0.05, Fig. 7b) and rearing (*p* > 0.05, Fig. 7c).

## Discussion

In this study we demonstrated the first evidence in vivo to show that oral and topical curcumin administration slows the progression of post-traumatic OA in the DMM mouse model. Specifically, we showed that oral or topical administration of curcumin immediately after DMM significantly slowed or delayed the initiation and progression of OA in mice. This was indicated by less cartilage erosion and proteoglycan loss, reduced synovitis and subchondral plate thickness, reduced degradation of type II collagen and aggrecan, and lower expression of MMP-13 and ADAMTS5 following curcumin treatment compared to vehicle controls. The preventative and therapeutic potential of curcumin is extremely valuable, given about 50 % of patients who suffer joint injuries, such as anterior cruciate ligament tears, develop OA within 10–15 years [47], and that there is no disease-modifying therapy for OA [48].

Furthermore, we provide the first evidence of a palliative effect of curcumin encapsulated in custom-made nanoparticles applied topically to an osteoarthritic joint in mice. Mice with DMM treated with curcumin nanoparticles exhibited decreased sensitivity to mechanical stimuli and increased locomotor behavior (i.e., distance traveled and rearing) compared to vehicle-treated mice, suggesting an improvement in OA-related pain. The results are consistent with a recently randomized, double-blind, placebo-controlled trial, in which patients with OA receiving a curcuminoid had significantly lower scores on the Western Ontario and McMaster Universities Osteoarthritis Index (WOMAC) and Lequesne’s pain functional index than patients receiving a placebo [23].

There is currently no cure for OA or a therapeutic agent with proven evidence to slow or halt the progression of OA [49]. Treatments used to temporarily relieve pain in OA, such as NSAIDs, may also cause severe gastrointestinal, renal, and cardiovascular side effects after long-term use [49–51]. In addition, patients experiencing pain relief without a concurrent improvement in the disease itself may become less conscientious about protecting their diseased joints (such as by avoiding overuse), and may unknowingly exacerbate the progression of OA. On the other hand, an OA drug that halts the progression of OA but does not relieve OA-related pain may not be effective, as patient compliance would likely be low. Upon further validation in other animal models and clinical trials, the effects of curcumin in both disease and symptom modification make it an attractive potential therapeutic agent for OA.

While the etiologic and pathogenic mechanisms for both initiation and progression of OA are not clear, inflammation, over-activated catabolic activity and oxidative stress responses are considered to be common in both processes [2, 44, 52, 53]. The effects of curcumin on attenuating inflammation, formation of reactive oxygen species, and catabolic activity have been suggested in chondrocytes in vitro [7, 14–16, 18, 19], in human synovial fibroblasts and in collagen-induced arthritis in mouse models [7, 14–16, 18, 19]. Furthermore, Colitti et al have shown an anti-inflammatory effect of curcumin on the gene expression of peripheral white blood cells in dogs with OA [16]. Consistent with these studies, we demonstrated that curcumin, in both the non-encapsulated (Fig. 1) and encapsulated forms (Fig. 3) exerts broad chondroprotective effects in human primary chondrocytes by suppressing the expression of genes encoding inflammatory cytokines IL-1β and TNF-α, and cartilage-degrading enzymes from the MMP family, including MMP-1, MMP-3, and MMP-13. We also demonstrated for the first time that curcumin suppresses expression of aggrecanase ADAMTS5, a key proteinase in cartilage destruction during OA that primarily cleaves the aggrecan components of the cartilage extracellular matrix [54–56]. Curcumin also induces gene expression of CITED2, an MMP-repressing transcriptional regulator. We previously demonstrated that CITED2, in response to moderate mechanical loading, represses expression of MMP-1 and MMP-13 in vitro [57] and in vivo [26]. NF-kB is a key factor that triggers the expression of various genes implicated in cartilage destruction, synovial membrane inflammation, and bone resorption [58, 59]. As CITED2 may negatively regulate NF-kB activity in embryonic kidney cells [60], curcumin may exert its chondroprotective effects by suppressing NF-kB activity by upregulating CITED2.

It has been reported that curcumin is barely soluble in water and poor absorption is attained from the epithelial cells in the gastrointestinal tract. Rats given an oral dose of curcumin excreted 75 % in the feces unchanged, with less than 0.02 % recovered from the liver, kidney, and body fat [61]. However, several studies analyzing plasma levels of curcumin or its metabolites have detected curcumin, although only small amounts, following relatively high doses of oral administration in humans [62, 63]. In this study, we demonstrated that oral administration of curcumin exerted efficacy in slowing the progression of post-traumatic OA. However, a palliative effect was not observed in mice with OA induced by DMM when curcumin was administrated orally in this study. The data suggest orally delivered curcumin is unlikely to reach biologically/pharmacologically active concentrations in the serum, synovial fluid, or joint tissues, that are sufficient to mitigate OA-related pain [4]. Together, our observation further indicates that relieving pain and its symptoms may require higher levels of curcumin compared to those required for disease modification.

As topical administration is a patient-friendly drug delivery method in OA treatment, we examined the efficacy of topical administration of nanoparticles encapsulating curcumin in OA disease modification and symptom improvement in mice with OA induced by DMM. Topical application of curcumin nanoparticles was efficacious not only in OA disease modification (Fig. 5), but also in relieving OA-related pain (Fig. 7). The data indicate that the topical application of curcumin encapsulated within nanoparticles preserves the chondroprotective activity of curcumin, and may increase its bioavailability.

Pathological changes in DMM-induced OA, including cartilage destruction, synovitis, and subchondral bone thickening, are observed in human OA [32]. Our study shows that curcumin treatment via oral (Fig. 2) or topical administration (Fig. 5) significantly improved OA-related pathological changes in the synovium and subchondral bone, indicating that curcumin has comprehensive potential for the treatment of joint tissues in OA [2].

The IPFP is an adipose tissue located within the knee joint synovial capsule, which may contribute to low-grade inflammation and cartilage degeneration through the secretion of adipokines and pro-inflammatory mediators into the synovial joint [64, 65]. In this study, we demonstrated that topically applied curcumin was largely localized in the infrapatellar fat pad (Fig. 4a). We further demonstrated that this treatment led to reduced expression of adipokines and pro-inflammatory mediators in the fat pad (Fig. 4b). These data suggest curcumin may slow the disease progression in OA, at least in part, by mitigating the pro-inflammatory mediating effect of the IPFP on cartilage and articular joints.

In this study, we provide the first evidence to demonstrate the efficacy of curcumin in OA disease and symptom modification using a post-traumatic OA mouse model. In addition to traumatic joint injuries, other conditions such as mechanical overuse and aging are risk factors for OA [66, 67]. Evaluating the efficacy of curcumin in other relevant OA models such as overuse-induced OA and spontaneous OA, which represents age-related OA, will be of interest.

## Conclusions

Using a post-traumatic OA mouse model, we provide the first evidence that curcumin has significant efficacy in slowing OA disease progression and a substantial effect on pain relief. Curcumin may exert its efficacy by regulating a broad spectrum of molecules including predominant proteinases in cartilage breakdown such as collagenase MMP-13, and aggrecanase ADAMTS5 in chondrocytes. The chondroprotective effects of curcumin, when administered topically, act through, at least in part, the suppression of relevant adipokines and other pro-inflammatory mediators that are critical for cartilage homeostasis in the infrapatellar fat pad.

## Abbreviations

ADAMTS, a disintegrin and metalloproteinase with thrombospondin motifs; ANOVA, analysis of variance; Cebpa, CCAAT/enhancer binding protein alpha; CITED2, Cbp/p300 interacting transactivator with ED-rich tail 2; Col2a1, collagen 2a1; COX-2, cyclooxygenase-2; DMEM, Dulbecco’s modified Eagle’s medium; DMM, destabilization of the medial meniscus; H&E, hematoxylin and eosin; IL-1β, interleukin-1 beta; IPFP, infrapatellar fat pad; MMP, matrix metalloproteinase; MMTL, medial meniscotibial ligament; NF-kB, nuclear factor kappa B; NSAID, non-steroidal anti-inflammatory drug; OA, osteoarthritis; OARSI, Osteoarthritis Research Society International; PBS, phosphate-buffered saline; PGE_2_, prostaglandin E2; Pparg, peroxisome proliferator-activated receptor gamma; TMOS, tetra-methyl-orthosilicate; TNF-α, tumor necrosis factor-alpha.

## Additional file

Additional file 1: Figure S1. Non-encapsulated curcumin reduces MMP-13 mRNA expression in a dose-and time-dependent manner. Human primary chondrocytes were treated with curcumin at indicated concentrations (0–200 μM) for 6 hours (**A**) or treated with curcumin at 100 μM at different durations (0–48 hours) (**B**) in the presence of IL-1β. **P* <0.05, *t* test or one-way ANOVA with Tukey post-hoc test, n = 3/group. (DOCX 116 kb)
