# Supplementary material for: Curcumin slows osteoarthritis progression and relieves osteoarthritis-associated pain symptoms in a post-traumatic osteoarthritis mouse model
Source: Arthritis Res Ther. 2016 Jun 3;18:128. doi: 10.1186/s13075-016-1025-y (PMC4891896; doi:10.1186/s13075-016-1025-y)
Supplement: Additional file 1: Figure S1. — Non-encapsulated curcumin reduces MMP-13 mRNA expression in a dose-and time-dependent manner. Human primary chondrocytes were treated with curcumin at indicated concentrations (0–200 μM) for 6 hours (A) or treated with curcumin at 100 μM at different durations (0–48 hours) (B) in the presence of IL-1β. *P <0.05, t test or one-way ANOVA with Tukey post-hoc test, n = 3/group. (DOCX 116 kb) [file 13075_2016_1025_MOESM1_ESM.docx]

**
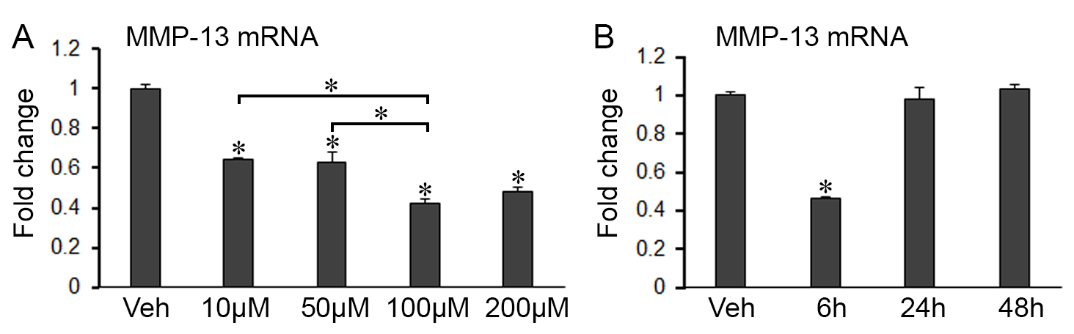
**

**Figure S1. Non-encapsulated curcumin reduces MMP-13 mRNA expression in a dose (A) and time (B) -dependent manner.** Human primary chondrocytes were treated with curcumin at indicated concentrations (0-200µM) for 6 hours (A) or treated with curcumin at 100 µM at different durations (0-48 hours) (B) in the presence of IL-1β. *p<0.05, t-test or one-way ANOVA with Tukey post-hoc test, n=3/group.
